# A Rare Case of Solitary Plasmacytoma Arising From the Sternum

**DOI:** 10.7759/cureus.23819

**Published:** 2022-04-04

**Authors:** Nyan Bethel, Henna Asrar, Jenna Dacosta, Andreas Savopoulos, Hamid Shaaban

**Affiliations:** 1 Internal Medicine, Saint Michael's Medical Center, Newark, USA; 2 Hematology and Oncology, Saint Michael's Medical Center, Newark, USA

**Keywords:** multiple myeloma, sternum, plasma cells, solitary bone plasmacytoma, extramedullary plasmacytoma, solitary plasmacytoma

## Abstract

Solitary plasmacytoma (SP) is characterized by an accumulation of neoplastic monoclonal plasma cells in a localized fashion, without evidence of multiple myeloma. It makes up <5% of all plasma cell neoplasms and is typically found in regions like the pelvis, ribs, vertebra, and spine. SP is classified into extramedullary plasmacytoma (EMP), which primarily affects soft tissues, and solitary bone plasmacytoma (SBP), which primarily affects the pelvis, ribs, vertebrae, and spine. We report a case of a 66-year-old man with sternal plasmacytoma presenting as chest pain. He was treated with radiation therapy. Here, we aim to describe the clinical features, diagnostic methods, treatment, and potential outcome in a patient with SBP.

## Introduction

Solitary plasmacytoma (SP) is a rare form of plasma cell neoplasm that is characterized by monoclonal plasma cell accumulation, without evidence of systemic myelomatosis [1-3]. Diagnosis of SBP requires a biopsy of the solitary bone lesion showing infiltration of plasma cells, negative results on a skeletal survey scan, negative bone marrow biopsy for clonal cells, and the absence of systemic findings suggestive of multiple myeloma which include hypercalcemia, renal failure, anemia, and osteolytic bone lesions [4-8]. Two-thirds of patients with SBP are males at the median age of 55. Usually, the initial presentation is pain at the affected site [9,10], but patients may experience symptoms due to mass effect, for example, a lesion on the vertebra may present with symptoms corresponding to nerve root or spinal cord compression. For SBP, radiotherapy is the treatment of choice [11-13]. Symptomatic relief, radiographic evidence of regression, and clinical stability are achieved in approximately 90% of all cases treated with radiotherapy [14,15]. Here, we present a case of sternal plasmacytoma in a male presenting sternal pain.

## Case presentation

A 66-year-old Hispanic male with a history of type II diabetes mellitus, hypertension, and hyperlipidemia presented to the emergency department with worsening sharp sternal pain with no aggravating or relieving factors over two weeks. On physical exam, his vital signs were unremarkable and there was tenderness to palpation over the mid sternal region with a palpable mass. The rest of his exam was unremarkable.

The initial laboratory test results were within the normal range (Tables 1, 2). The troponins and EKG were unremarkable.

**Table 1:** Key laboratory values

Test	Reference Value	Result
BUN	6-24 (mg/dL)	23
Creatinine	0.6-1.2 (mg/dL)	0.9
Alkaline Phosphatase	40-115 (U/L)	86
Total Protein	6.4-8.4 (g/dL)	7.4
Albumin	3.6-5.1 (g/dL)	3.9
Calcium	8.6-10.4 (mg/dL)	9.1

**Table 2:** Hemogram test values

Test	Reference Value	Result
WBC	4.4-11 (10*3µ/L)	5.3
Hemoglobin	13.5-17.5 (g/dL)	13.5
Hematocrit	38-50 (%)	39.5
Platelets	150-450 (10*3µ/L)	268

**Figure 1:**
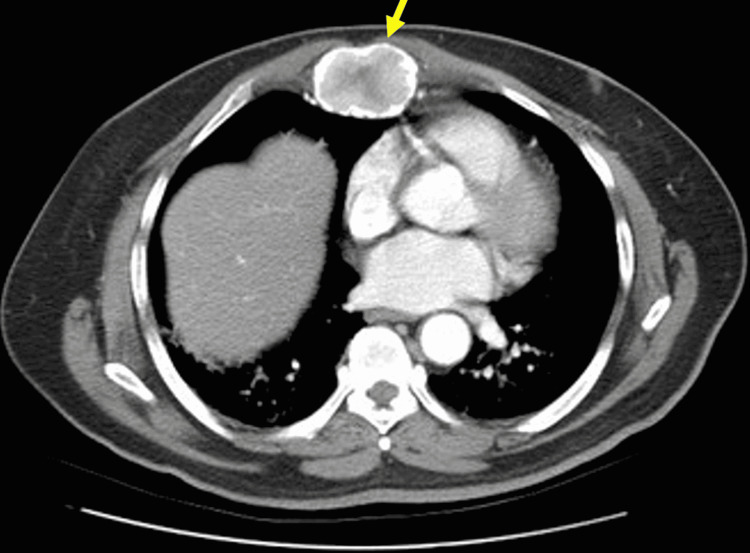
Axial computed tomography (CT) image of an osteolytic sternal mass

**Figure 2:**
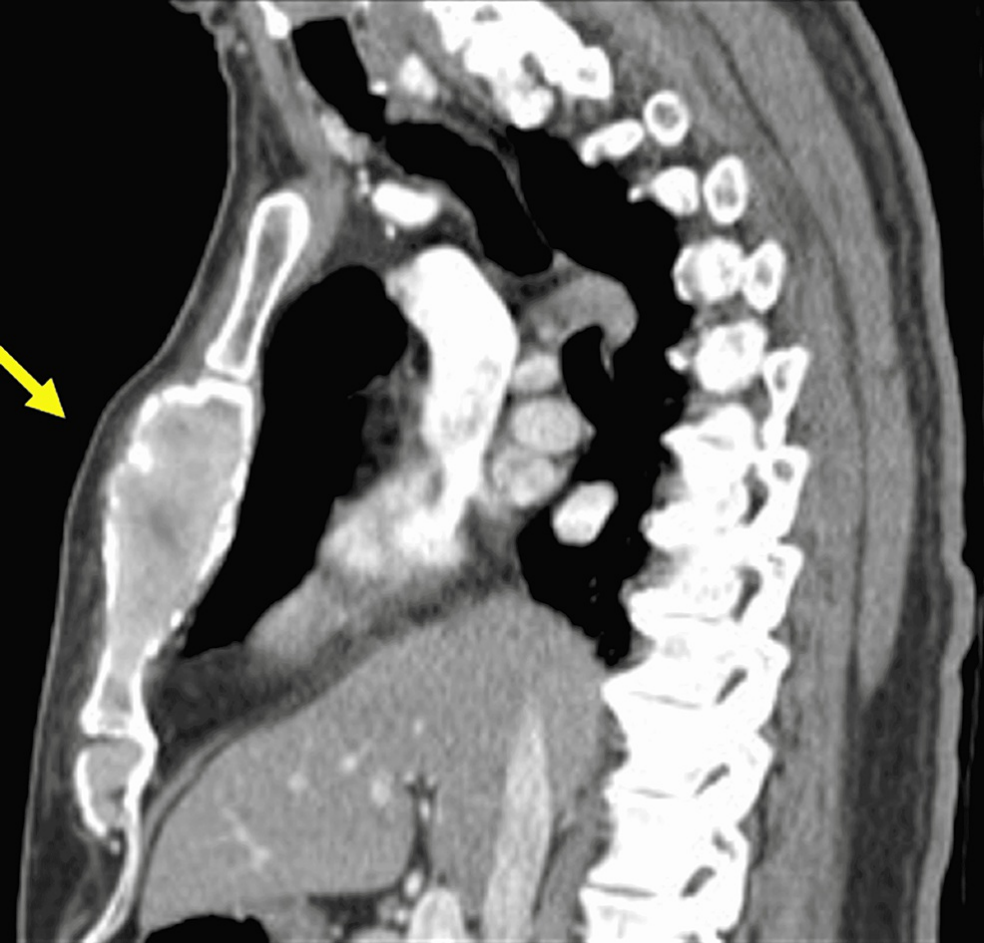
Sagittal computed tomography view of the sternal mass

A chest X-ray revealed no signs of acute cardiopulmonary pathology. A chest CT scan (Figures 1, 2) revealed an expansile sternal mass measuring 3x9 cm.

**Figure 3:**
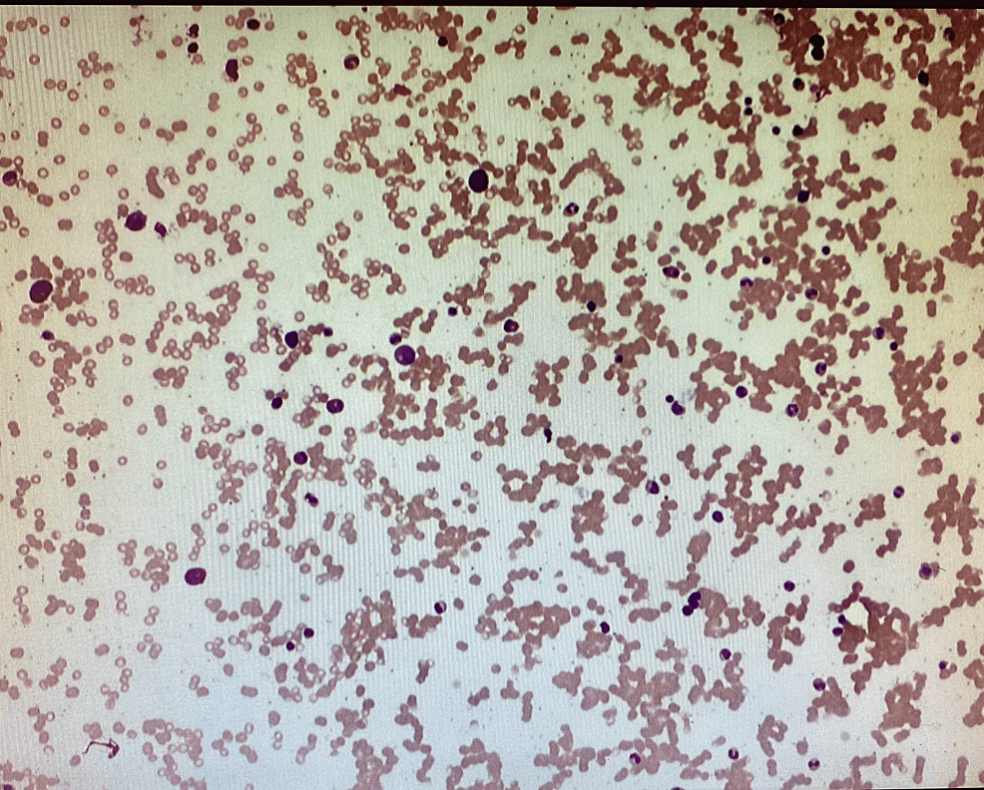
Immunohistochemical stain (CD138) on sternal biopsy specimen revealing plasma cells (60x)

**Figure 4:**
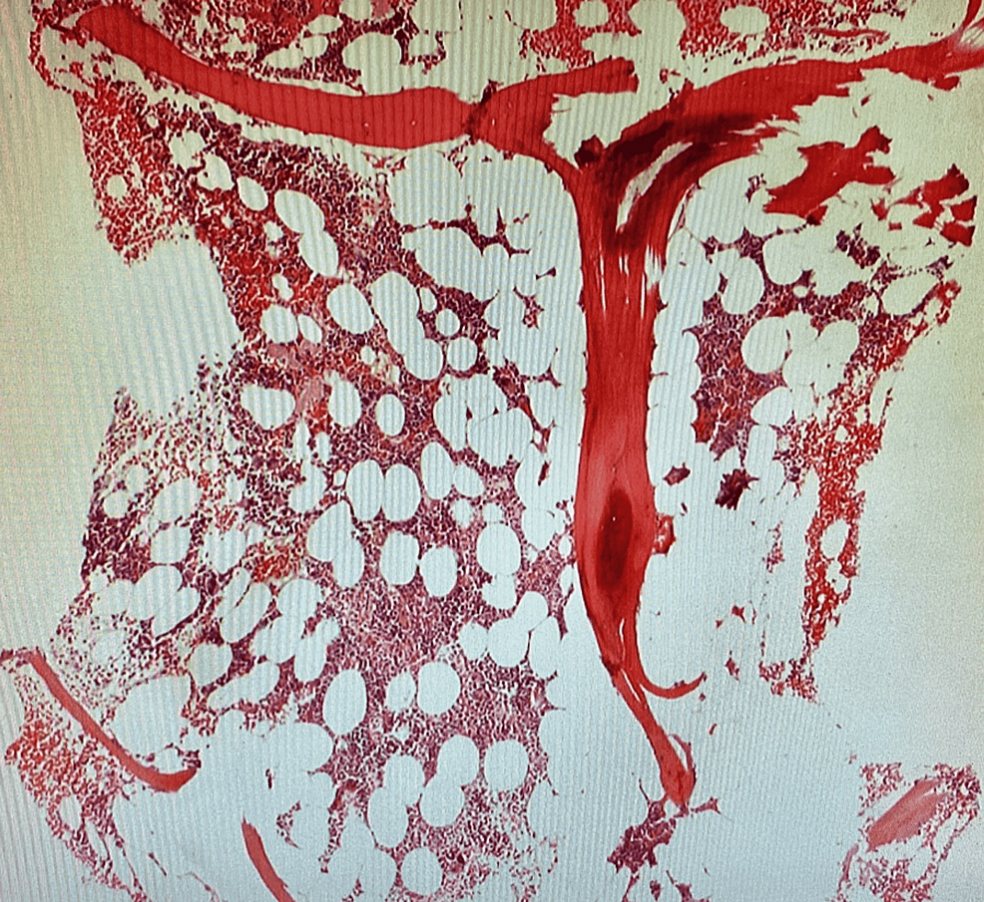
Bone marrow biopsy revealed minimal marrow involvement (H&E stain, 60x)

A skeletal bone survey scan revealed no osteolytic or osteoblastic lesions. The sternal bone biopsy was done and histopathology revealed sheets of atypical cells with no obvious hematopoiesis. Immunohistochemical staining revealed that CD138+ plasma cells account for more than 95% of total cellularity with a lambda clonal phenotype and abnormal CD56 co-expression (Figure 3). There was no evidence of heavy immunoglobulin chain expression. Pancytokeratin (AE1/AE3) was absent. A right posterior iliac crest bone marrow biopsy was then performed on the patient to rule out multiple myeloma. The histopathology of that biopsy revealed normocellular marrow with unremarkable trilineage hematopoiesis and 5%-10% of plasma cells with slight lambda predominance (Figure 4). Immunohistochemical stains of the bone marrow further showed plasma cells that were stained positive for CD138+, and a smaller subset staining positive for CD56. They were negative for CD117 and CyclinD1. The serum protein electrophoresis with immunofixation and quantitation of immunoglobulins, and a serum-free light chain assay were all normal. The urinalysis, 24h urine collection for proteinuria, electrophoresis, and immunofixation did not reveal any abnormalities. These blood tests were essentially done to rule out active myeloma.

Taking into consideration the clinical presentation, imaging findings, and biopsy results, a diagnosis of solitary sternal plasmacytoma with minimal marrow involvement was made. The patient was referred to the Radiation Oncologist and he received a total of 25 sessions of radiation therapy. Of that, 16 of those sessions were conducted at a dose of 28 Gy and the remaining at 45 Gy. At this time, he experienced symptomatic relief. He will continue to follow up with his oncologist for scans to monitor for radiologic regression in the sternal mass as well as evidence of progression to multiple myeloma.

## Discussion

On the spectrum of plasma cell dyscrasias, plasmacytomas account for only 5% of plasma cell neoplasms [6,7]. Further divided based on location, a solitary bone plasmacytoma (SBP) occurs in red marrow containing bone, often vertebrae in the axial skeleton, whereas extramedullary plasmacytomas (EMPs) are found in soft tissue [2-5]. SBPs favor a poor prognosis due to an increased risk of progression to multiple myeloma in comparison to its counterpart, EMP. With an 85% risk of progression at 10 years and nearly 100% at 15 years, the median time of SBP progression to MM is approximately 2-3 years despite treatment [2,12-18].

The most common presenting symptom of an SBP is bone pain [11-13]. Depending on its location, it may also present with signs of compression neuropathy and pathologic fractures.

According to the International Myeloma Working Group (IMWG), in order to make a definitive diagnosis of SBP, the following criteria must be met: skeletal survey modality confirming the solitary bone lesion, tissue biopsy confirming plasma cell infiltration, and bone marrow biopsy showing no or minimal infiltration (<10% plasma cells) [2]. To diagnose SBP, multiple myeloma must also be excluded. Therefore, to confirm the diagnosis of SBP, there must be the absence of any of the “CRAB” symptoms of multiple myeloma (hypercalcemia, renal insufficiency, anemia, bone lesions) [3,14].

Skeletal radiographs are only 30% sensitive in identifying early lytic bone lesions and extraosseous lesions, therefore they are not the gold standard for the diagnostic imaging evaluation of plasmacytomas [2]. Instead, there is a role for CT, MRI, and PET/ CT imaging modalities. CT scans are able to elucidate nerve compressions; however, MR imaging is typically more ideal to show extraosseous lesions. The CT scan is able to become more specific when used in conjunction with PET. Our patient did not get a PET because of insurance and financial issues. In one study, it was shown that CT scans alone provide a specificity of 89% compared to 99% when used in conjunction with 18F-FDG PET [17]. Staging of the tumor is often accomplished using MRI modality, appearing hypo/iso-intense on T1- weighted images and hyperintense on T2- weighted images [17,18].

For patients with suspected SBP, a bone marrow biopsy, as well as aspiration, is recommended. By using either immunophenotyping via flow cytometry, kappa/lambda chain labeling of the bone marrow aspirate sample, or immunohistochemistry of the biopsy, the degree of plasma cell infiltration can be better determined [15,16]. This becomes important in not only the diagnosis of SBP but also in prognosis [2-9]. One study found that the progression-free survival (PFS) in patients with and without bone marrow infiltration confirmed by flow cytometry, was 15% and 42%, respectively [15]. In another study, 71% of SBP patients with evidence of infiltration progressed to MM within 26 months versus only 8% of SBP patients without bone marrow involvement [16].

There are several other prognostic factors to consider in a patient with SBP. Persistent elevation in monoclonal serum protein, despite treatment, has shown an increased risk of progression to MM [13]. Although for some patients, levels may remain elevated for the first year after initiating treatment before declining [13]. In one study, a 10-year PFS of 29% versus 91% in SBP patients illustrates the prognostic difference in patients with persistently elevated serum monoclonal protein after treatment (radiotherapy in this case) to those with resolving protein levels, respectively [10]. In addition, some studies have shown that a post-treatment protein level >0.5 g/dL is associated with a negative prognosis. It should also be noted that the presence and quantity of serum monoclonal proteins at diagnosis yields no prognostic information [13,16,19].

Other prognostic factors linked to a poor outcome include age >40 years, tumor size >4-5cm, higher histological grade, and degree of angiogenesis [17]. A higher degree of angiogenesis in anaplastic type plasmacytomas is correlated with higher progression rates to MM. In a 2003 study, nearly 65% of plasmacytoma biopsy samples with high-grade angiogenesis likely progressed to MM and had a shorter PFS [17]. Research strongly supports the prognostic value of a serum-free light chain (SFLC) ratio. It is an independent predictor of prognosis when outside the normal range (<0.26 or >1.65) and has been implemented in prognostic scoring models. Fouquet et al proposed that an abnormal SFLC value and the presence of at least two hypermetabolic lesions on imaging (specifically PET/CT) at diagnosis were sufficient predictors for early progression to MM [19].

The standard treatment for SBP is radiotherapy [4,9]. Although there is no guideline for a specific regimen, RT doses of 40-50Gy are commonly used over the course of four weeks [5,18]. The consensus in the literature supports a local control rate of over 80% in the treatment of SBP with RT. The treatment field should ideally include the lesions, as identified by imaging, as well as healthy tissue margins of at least 2cm. If spinal lesions are present, radiotherapy should also be targeted to at least one uninvolved vertebra on either side [5].

The role of surgery is debatable. While some literature favors a more conservative approach to surgery, others argue that surgery combined with RT is necessitated for optimal treatment as it reduces tumor burden, especially in the management of EMP as it allows for larger masses to be resected. Commonly, however, surgery is reserved for fracture fixation, neural decompression, and spine stabilization only [5-7].

The use of chemotherapy in the treatment of SBP is controversial. Although it provides no benefit as a standalone treatment as RT does, it may provide a survival advantage when used as an adjuvant to RT. It may be used in high-risk patients (ie, tumor size >5cm, the persistence of monoclonal proteins) or for patients refractory to RT since it slows progression to MM even though it does not affect incidence [10-13]. In one study, SBP patients on a three-year course of melphalan and prednisone delayed MM progression from 29 to 59 months [2].

Despite excellent responses to RT, a majority of patients with SBP usually progress to MM [17,18]. These patients should be followed up with imaging, laboratory evaluation electrophoresis, and immunofixation; preferably the same mode as used for diagnosis to monitor for progression to MM.

## Conclusions

SP is a distinct entity that can occur as SBP or EMP. Our patient had an initial presentation of chest pain and was subsequently diagnosed with solitary sternal plasmacytoma. Healthcare providers should pay close attention to elderly patients with similar clinical presentations because early detection of SP may lead to earlier treatment, and closer monitoring with serum electrophoresis and immunofixation to avoid or intervene sooner if there is a potential progression into myeloma.
